# Unravelling similarities and differences in the role of circular and linear *PVT1* in cancer and human disease

**DOI:** 10.1038/s41416-021-01584-7

**Published:** 2021-11-09

**Authors:** Debora Traversa, Giorgia Simonetti, Doron Tolomeo, Grazia Visci, Gemma Macchia, Martina Ghetti, Giovanni Martinelli, Lasse S. Kristensen, Clelia Tiziana Storlazzi

**Affiliations:** 1 Department of Biology, University of Bari “Aldo Moro”, Bari, Italy; 2 IRCCS Istituto Romagnolo per lo Studio dei Tumori (IRST) “Dino Amadori”, Meldola, FC Italy; 3 Department of Biomedicine, Aarhus University, Aarhus, Denmark

**Keywords:** Cancer genetics, Tumour biomarkers

## Abstract

The plasmacytoma variant translocation 1 (*PVT1*) is a long non-coding RNA gene involved in human disease, mainly in cancer onset/progression. Although widely analysed, its biological roles need to be further clarified. Notably, functional studies on *PVT1* are complicated by the occurrence of multiple transcript variants, linear and circular, which generate technical issues in the experimental procedures used to evaluate its impact on human disease. Among the many *PVT1* transcripts, the linear PVT1 (lncPVT1) and the circular hsa_circ_0001821 (circPVT1) are frequently reported to perform similar pathologic and pro-tumorigenic functions when overexpressed. The stimulation of cell proliferation, invasion and drug resistance, cell metabolism regulation, and apoptosis inhibition is controlled through multiple targets, including MYC, p21, STAT3, vimentin, cadherins, the PI3K/AKT, HK2, BCL2, and CASP3. However, some of this evidence may originate from an incorrect evaluation of these transcripts as two separate molecules, as they share the lncPVT1 exon-2 sequence. We here summarise lncPVT1/circPVT1 functions by mainly focusing on shared pathways, pointing out the potential bias that may exist when the biological role of each transcript is analysed. These considerations may improve the knowledge about lncPVT1/circPVT1 and their specific targets, which deserve further studies due to their diagnostic, prognostic, and therapeutic potential.

## Background

The one-way DNA–RNA–protein paradigm has been outdated since the discovery of non-coding RNA (ncRNA) genes, which account for two-thirds of the total number of human genes [1]. ncRNAs are involved in critical biological processes. They can regulate gene expression at the transcriptional and post-transcriptional levels and are often deregulated in a variety of human diseases [2]. Thus, they may represent potential keystones for the new targeted therapies of incurable diseases, including cancer.

ncRNAs are classified into two subcategories: small and long ncRNAs (lncRNAs), of less and more than 200 nt, respectively [3]. To date, despite the identification of many lncRNAs, most of them still need to be functionally characterised.

Circular RNAs (circRNAs) represent a particular subtype of ncRNAs originating from back-splicing events. Discovered 40 years ago, they initially were considered as splicing by-products with unknown functions [4]. Their roles have recently been re-evaluated due to the discovery of thousands of circRNA entities. Some of them are highly abundant, evolutionary conserved, and involved in cellular differentiation and tissue homoeostasis, as well as in the development of multiple diseases [4, 5]. Notably, the majority of circRNAs originate from genes that show oncogenic effects [6].

Great interest has recently been devoted to the “plasmacytoma variant translocation 1” (*PVT1*) lncRNA gene, which produces both linear and circular transcripts that have been reported to be overexpressed in several cancer types [7]. Interestingly, positive correlations between *PVT1* overexpression and tumour progression are frequently observed [8–10] (see the section “Clinical impact of lncPVT1 and circPVT1”).

**Fig. 1:**
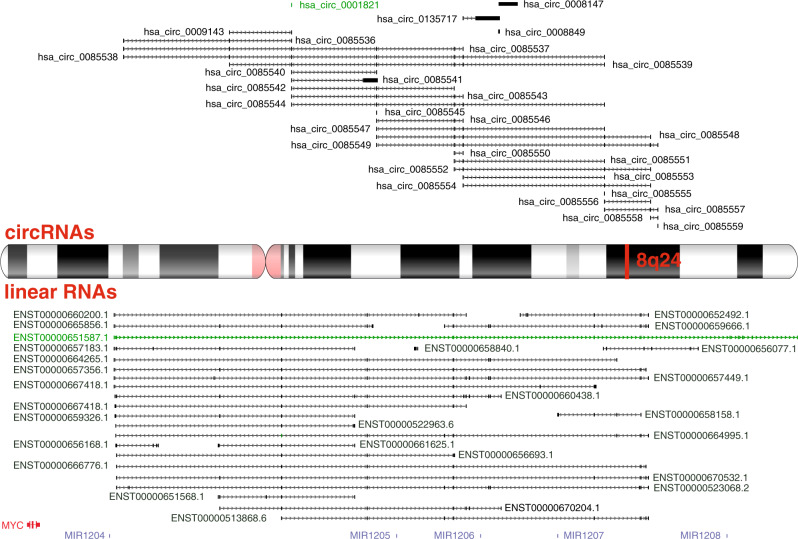
The *PVT1* genomic locus and its circular and linear transcript isoforms. Chromosome 8 ideogram. The circular (from the CircInteractome and circBase databases) and linear transcripts (from the UCSC Genome Browser) of *PVT1* are indicated at the top and bottom parts of the figure, respectively. Each transcript isoform is represented at its correspondent map position on the genome (GRCh38/hg38). The most studied RNA entities are shown in green. *MYC* (red) and miRNA genes (light purple) are also represented.

*PVT1* maps at the 8q24 chromosomal band, reported as a gene desert, harbouring two fragile sites (FRA8C and FRA8D) [11]. It is an exceptionally complex locus, which gives rise to 176 linear splicing variants (according to the Ensembl Genome Browser, https://www.ensembl.org/index.html), 27 of which are also reported at the UCSC Genome Browser (https://genome.ucsc.edu/index.html, Fig. 1), as well as to 29 circular RNAs, as reported in the CircInteractome (https://circinteractome.nia.nih.gov/) [12], and circBase (http://www.circbase.org/) [13] databases (Table 1). In addition, according to the UCSC Genome Browser, the *PVT1* locus harbours five highly conserved microRNAs (miRNAs) (Fig. 1). Some linear transcripts were detected by exon-specific RT-qPCR [14], 5’RACE PCR [8], and lncRNA microarrays [15]; others resulted from transcript predictions by computational approaches. The most extended linear transcript at the *PVT1* locus is the PVT1-224/ENST00000651587.1 isoform (herein referred to as lncPVT1). A few reports have addressed the differential roles of such linear and circular transcript variants in cancer and disease so far. For the linear isoforms, the overexpression of different splicing variants was observed in ovarian cancer cell lines [8], gastrointestinal tumours [16], and prostate cancer [14, 17]. For instance, in colorectal cancer (CRC), He et al. identified the overexpression of 14 *PVT1* lncRNAs in CRC samples compared with paired adjacent non-tumour tissues using lncRNA microarray [15]. In particular, they focused on the PVT1-214 variant, the most overexpressed one, revealing its role in the upregulation of the Lin28 RNA-binding protein by acting at both transcript (competing with miR-128 for the *Lin28* mRNA binding) and protein level. Furthermore, the authors indicated an effect on let-7 miRNA expression, offering a new scenario where the PVT1-214/Lin28/let-7 axis serves as a critical regulator of CRC pathogenesis [15]. Moreover, Martínez-Barriocanal et al. reported a role for 11 *PVT1* splicing variants as miRNA sponges in gastrointestinal tumours [16] (Table 1).

**Table 1:** lncPVT1 and circPVT1 transcript variants.

Name	Transcript ID	Exon no.	Size (bp)	Position (GRCh38/hg38)	Reference (identification)	Reference (function)	Name	Transcript ID	Exon no.	Size (bp)	Position (GRCh38/hg38)	Reference (identification)	Reference (function)
**lncPVT1**							**lncPVT1**						
PVT1-201	ENST00000504719.7	4	1017	chr8: 127,794,535–127,932,706	10.1038/s41388-018-0432-8, 10.3389/fonc.2020.00038	10.1038/s41388-018-0432-8, 10.3389/fonc.2020.00038	PVT1-304	ENST00000661830.1	7	1490	chr8: 127,794,533–128,101,256	–	–
PVT1-202	ENST00000512617.7	6	1109	chr8: 127,984,004–128,101,256	–	–	PVT1-305	ENST00000661988.1	6	1580	chr8: 127,795,413–127,996,670	–	–
PVT1-203	ENST00000513868.6	8	1699	chr8: 127,890,628–128,101,253	–	–	PVT1-306	ENST00000662061.1	7	1139	chr8: 127,794,590–127,996,670	–	–
PVT1-204	ENST00000517525.2	10	1194	chr8: 127,794,538–127,996,670	–	–	PVT1-307	ENST00000662410.1	11	1668	chr8: 127,794,560–128,101,256	–	–
PVT1-205	ENST00000517790.2	4	1513	chr8: 127,795,209–127,996,670	10.1038/s41388-018-0432-8, 10.3389/fonc.2020.00038	10.1038/s41388-018-0432-8, 10.3389/fonc.2020.00038	PVT1-308	ENST00000662413.1	9	1727	chr8: 127,794,565–128,101,256	–	–
PVT1-206	ENST00000517838.6	4	821	chr8: 127,890,587–127,996,670	10.1038/s41388-018-0432-8, 10.3389/fonc.2020.00038	10.1038/s41388-018-0432-8, 10.3389/fonc.2020.00038	PVT1-309	ENST00000662709.1	5	858	chr8: 127,984,004–128,101,256	–	–
PVT1-207	ENST00000518528.2	4	995	chr8: 127,795,139–127,932,701	10.1038/s41388-018-0432-8, 10.3389/fonc.2020.00038	10.1038/s41388-018-0432-8, 10.3389/fonc.2020.00038	PVT1-309	ENST00000662709.1	5	858	chr8: 127,984,004–128,101,256	–	–
PVT1-208	ENST00000519481.6	6	1010	chr8: 127,854,398–127,996,670	10.1038/s41388-018-0432-8, 10.3389/fonc.2020.00038	10.1038/s41388-018-0432-8, 10.3389/fonc.2020.00038	PVT1-312	ENST00000663461.1	7	1471	chr8: 127,890,601–128,101,252	–	–
PVT1-209	ENST00000520913.2	4	835	chr8: 127,854,396–127,932,696	10.1038/s41388-018-0432-8, 10.3389/fonc.2020.00038	10.1038/s41388-018-0432-8, 10.3389/fonc.2020.00038	PVT1-312	ENST00000663461.1	7	1471	chr8: 127,890,601–128,101,252	–	–
PVT1-210	ENST00000521122.2	3	1578	chr8: 127,854,396–127,932,708	–	–	PVT1-313	ENST00000663715.1	3	615	chr8: 128,045,230–128,101,256	–	–
PVT1-211	ENST00000521600.5	4	408	chr8: 127,989,217–128,096,578	–	–	PVT1-314	ENST00000663753.1	7	1206	chr8: 127,794,559–128,101,256	–	–
PVT1-212	ENST00000521951.1	3	1535	chr8: 127,794,557–127,940,454	–	–	PVT1-315	ENST00000664139.1	4	1036	chr8: 127,794,531–127,932,708	–	–
PVT1-213	ENST00000522414.2	4	1132	chr8: 128,049,388–128,099,890	10.1038/s41388-018-0432-8, 10.3389/fonc.2020.00038	10.1038/s41388-018-0432-8, 10.3389/fonc.2020.00038	PVT1-316	ENST00000664214.1	3	861	chr8: 127,794,527–127,932,707	–	–
PVT1-214	ENST00000522875.5	8	922	chr8: 127,989,261–128,096,656	10.1038/s41388-018-0432-8, 10.3389/fonc.2020.00038	10.1038/s41388-018-0432-8, 10.3389/fonc.2020.00038	PVT1-317	ENST00000664265.1	7	1720	chr8: 127,794,563–128,083,335	–	–
PVT1-215	ENST00000522963.6	4	1619	chr8: 127,795,221–127,932,720	10.1038/s41388-018-0432-8, 10.3389/fonc.2020.00038	10.1038/s41388-018-0432-8, 10.3389/fonc.2020.00038	PVT1-318	ENST00000664293.1	6	1206	chr8: 127,794,559–127,996,670	–	–
PVT1-216	ENST00000523068.2	11	2255	chr8: 127,796,033–128,101,256	–	–	PVT1-319	ENST00000664299.1	7	1396	chr8: 127,795,808–127,996,670	–	–
PVT1-217	ENST00000523190.6	6	963	chr8: 128,009,614–128,101,256	10.1038/s41388-018-0432-8, 10.3389/fonc.2020.00038	10.1038/s41388-018-0432-8, 10.3389/fonc.2020.00038	PVT1-320	ENST00000664610.1	4	1114	chr8: 127,998,216–128,070,873	–	–
PVT1-218	ENST00000523328.6	5	1047	chr8: 127,794,565–127,932,709	–	–	PVT1-321	ENST00000664742.1	7	1200	chr8: 127,984,004–128,101,256	–	–
PVT1-219	ENST00000523427.2	2	837	chr8: 127,794,567–127,891,257	10.1038/s41388-018-0432-8, 10.3389/fonc.2020.00038	10.1038/s41388-018-0432-8, 10.3389/fonc.2020.00038	PVT1-322	ENST00000664924.1	3	886	chr8: 127,854,728–127,932,701	–	–
PVT1-220	ENST00000524165.6	4	1114	chr8: 127,794,557–127,932,710	–	–	PVT1-323	ENST00000664995.1	8	2230	chr8: 127,795,353–128,101,247	–	–
PVT1-221	ENST00000650846.1	4	1020	chr8: 128,120,269–128,187,031	–	–	PVT1-324	ENST00000665166.1	3	965	chr8: 127,854,635–127,932,701	–	–
PVT1-222	ENST00000650930.1	4	777	chr8: 128,045,230–128,101,256	–	–	PVT1-325	ENST00000665175.1	4	964	chr8: 128,082,378–128,101,256	–	–
PVT1-223	ENST00000651568.1	5	971	chr8: 127,854,400–127,932,712	–	–	PVT1-326	ENST00000665246.1	4	1096	chr8: 127,795,196–127,932,708	–	–
PVT1-224	ENST00000651587.1	11	2444	chr8: 127,794,541–128,187,101	–	–	PVT1-327	ENST00000665372.1	4	762	chr8: 127,794,565–128,101,256	–	–
PVT1-225	ENST00000651664.1	2	914	chr8: 127,890,226–127,932,712	–	–	PVT1-328	ENST00000665698.1	4	1000	chr8: 127,794,559–128,101,256	–	–
PVT1-226	ENST00000652492.1	6	1017	chr8: 128,027,509–128,101,099	–	–	PVT1-329	ENST00000665721.1	5	1162	chr8: 127,794,700–127,995,300	–	–
PVT1-227	ENST00000652695.1	7	1126	chr8: 127,859,787–127,996,670	–	–	PVT1-330	ENST00000665737.1	7	1410	chr8: 127,794,559–128,101,254	–	–
PVT1-228	ENST00000652728.1	3	812	chr8: 128,049,351–128,101,256	–	–	PVT1-331	ENST00000665856.1	5	1965	chr8: 127,794,538–127,943,442	–	–
PVT1-229	ENST00000652816.1	8	1610	chr8: 127,794,537–128,101,256	–	–	PVT1-332	ENST00000666039.1	3	520	chr8: 128,046,607–128,101,256	–	–
PVT1-230	ENST00000652883.1	5	821	chr8: 128,045,233–128,101,256	–	–	PVT1-333	ENST00000666076.1	3	1062	chr8: 127,984,004–127,995,613	–	–
PVT1-231	ENST00000652993.1	4	866	chr8: 128,046,371–128,101,256	–	–	PVT1-334	ENST00000666080.1	7	1481	chr8: 127,794,555–128,101,256	–	–
PVT1-232	ENST00000653406.1	2	619	chr8: 127,891,959–127,932,708	–	–	PVT1-335	ENST00000666105.1	3	608	chr8: 128,049,409–128,101,256	–	–
PVT1-233	ENST00000653497.1	7	1278	chr8: 127,994,949–128,101,254	–	–	PVT1-336	ENST00000666147.1	6	2097	chr8: 127,997,046–128,101,256	–	–
PVT1-234	ENST00000653522.1	6	1275	chr8: 127,794,527–128,101,256	–	–	PVT1-337	ENST00000666223.1	5	963	chr8: 128,049,409–128,101,256	–	–
PVT1-235	ENST00000653608.1	4	906	chr8: 128,049,367–128,101,256	–	–	PVT1-338	ENST00000666353.1	12	1312	chr8: 127,794,542–127,996,670	–	–
PVT1-236	ENST00000653845.1	6	1262	chr8: 127,794,538–128,101,256	–	–	PVT1-339	ENST00000666452.1	6	1001	chr8: 127,984,004–128,101,256	–	–
PVT1-237	ENST00000653853.1	5	1490	chr8: 127,984,004–127,995,613	–	–	PVT1-340	ENST00000666776.1	6	1628	chr8: 127,795,808–128,099,890	–	–
PVT1-238	ENST00000653990.1	7	1329	chr8: 127,983,878–128,101,256	–	–	PVT1-341	ENST00000666777.1	6	1555	chr8: 127,795,600–128,101,256	–	–
PVT1-239	ENST00000654091.1	5	1311	chr8: 127,794,565–127,999,334	–	–	PVT1-342	ENST00000666842.1	6	1019	chr8: 127,795,200–127,996,670	–	–
PVT1-240	ENST00000654105.1	9	1460	chr8: 127,794,683–128,101,256	–	–	PVT1-343	ENST00000666878.1	5	830	chr8: 128,045,332–128,101,256	–	–
PVT1-241	ENST00000654324.1	7	1411	chr8: 127,794,550–128,101,252	–	–	PVT1-344	ENST00000667149.1	5	1463	chr8: 127,795,932–127,942,997	–	–
PVT1-242	ENST00000654369.1	5	1482	chr8: 127,795,346–127,996,670	–	–	PVT1-345	ENST00000667204.1	4	897	chr8: 127,794,600–127,932,701	–	–
PVT1-243	ENST00000655099.1	5	855	chr8: 127,984,004–128,101,256	–	–	PVT1-346	ENST00000667305.1	9	1701	chr8: 127,794,526–128,101,256	–	–
PVT1-244	ENST00000655148.1	4	724	chr8: 128,049,409–128,101,256	–	–	PVT1-347	ENST00000667418.1	5	2143	chr8: 127,794,576–128,071,539	–	–
PVT1-245	ENST00000655594.1	2	978	chr8: 127,846,054–127,852,712	–	–	PVT1-348	ENST00000667539.1	5	1041	chr8: 127,794,555–127,996,670	–	–
PVT1-246	ENST00000655783.1	7	1118	chr8: 127,984,000–128,101,256	–	–	PVT1-349	ENST00000667630.1	2	1634	chr8: 128,095,298–128,101,256	–	–
PVT1-247	ENST00000656077.1	6	1109	chr8: 128,046,604–128,101,256	–	–	PVT1-350	ENST00000667714.1	7	1269	chr8: 127,794,675–128,101,255	–	–
PVT1-248	ENST00000656168.1	4	935	chr8: 127,795,409–127,820,382	–	–	PVT1-351	ENST00000668098.1	5	1985	chr8: 127,997,045–128,101,256	–	–
PVT1-249	ENST00000656396.1	5	938	chr8: 127,795,967–127,932,701	–	–	PVT1-352	ENST00000668123.1	7	1433	chr8: 127,7958,08–128,101,255	–	–
PVT1-250	ENST00000656402.1	8	1585	chr8: 127,794,590–128,101,256	–	–	PVT1-353	ENST00000668215.1	2	590	chr8: 128,049,400–128,096,758	–	–
PVT1-251	ENST00000656411.1	9	1281	chr8: 127,984,004–128,101,256	–	–	PVT1-354	ENST00000668351.1	3	759	chr8: 128,049,401–128,101,256	–	–
PVT1-252	ENST00000656491.1	7	1167	chr8: 127,794,609–127,996,670	–	–	PVT1-355	ENST00000668479.1	4	1091	chr8: 128,048,135-128,101,256	–	–
PVT1-253	ENST00000656532.1	7	1909	chr8: 127,795,358–127,996,670	–	–	PVT1-356	ENST00000668480.1	4	956	chr8: 128,049,409–128,101,256	–	–
PVT1-254	ENST00000656693.1	4	1990	chr8: 127,795,802–127,990,365	–	–	PVT1-357	ENST00000668619.1	3	1685	chr8: 128,047,339–128,101,256	–	–
PVT1-255	ENST00000656880.1	5	1017	chr8: 127,794,537–127,996,670	–	–	PVT1-358	ENST00000669082.1	5	1280	chr8: 127,854,500–127,996,670	–	–
PVT1-256	ENST00000656948.1	3	904	chr8: 127,854,709–127,932,706	–	–	PVT1-359	ENST00000669132.1	4	1156	chr8: 127,794,537–127,932,701	–	–
PVT1-257	ENST00000656999.1	6	1314	chr8: 127,998,029–128,101,255	–	–	PVT1-360	ENST00000669272.1	4	1417	chr8: 127,795,813–127,942,997	–	–
PVT1-258	ENST00000657112.1	5	1499	chr8: 127,794,559–128,083,366	–	–	PVT1-361	ENST00000669407.1	3	890	chr8: 127,916,559–127,943,001	–	–
PVT1-259	ENST00000657183.1	6	1172	chr8: 127,794,542–127,932,696	–	–	PVT1-362	ENST00000669416.1	3	1516	chr8: 127,795,149–127,932,710	–	–
PVT1-260	ENST00000657211.1	6	1353	chr8: 127,794,538–128,101,256	–	–	PVT1-363	ENST00000669509.1	5	1066	chr8: 128,049,378–128,101,256	–	–
PVT1-261	ENST00000657289.1	8	1581	chr8: 127,794,533–128,101,256	–	–	PVT1-364	ENST00000669951.1	7	1485	chr8: 127,795,561–127,996,667	–	–
PVT1-262	ENST00000657297.1	4	907	chr8: 127,794,559–127,932,701	–	–	PVT1-365	ENST00000670009.1	3	456	chr8: 128,049,400–128,101,256	–	–
PVT1-263	ENST00000657356.1	7	1697	chr8: 127,794,567–128,099,887	–	–	PVT1-366	ENST00000670204.1	7	1600	chr8: 127,855,209–128,017,216	–	–
PVT1-264	ENST00000657384.1	4	1068	chr8: 127,795,198–127,932,701	–	–	PVT1-367	ENST00000670223.1	6	1321	chr8: 127,794,537–128,101,256	–	–
PVT1-265	ENST00000657449.1	11	2149	chr8: 127,794,575–128,101,254	–	–	PVT1-368	ENST00000670532.1	9	1840	chr8: 127,795,926–128,101,256	–	–
PVT1-266	ENST00000657451.1	7	1454	chr8: 127,795,763–128,101,254	–	–	PVT1-369	ENST00000670535.1	4	876	chr8: 128,049,400–128,101,256	–	–
PVT1-267	ENST00000657517.1	2	1253	chr8: 127,795,535–127,852,712	–	–	PVT1-370	ENST00000670602.1	3	1124	chr8: 127,890,196–127,932,701	–	–
PVT1-268	ENST00000657596.1	3	1455	chr8: 127,795,346–127,932,700	–	–	PVT1-371	ENST00000670626.1	6	1114	chr8: 127,890,715–127,996,670	–	–
PVT1-269	ENST00000657667.1	6	1378	chr8: 127,795,754–128,101,256	–	–	PVT1-372	ENST00000670714.1	7	1756	chr8: 127,795,552–128,101,256	–	–
PVT1-270	ENST00000657682.1	3	611	chr8: 128,049,406–128,101,256	–	–	PVT1-373	ENST00000670795.1	5	1348	chr8: 127,794,561–127,995,232	–	–
PVT1-271	ENST00000657693.1	6	977	chr8: 127,794,555–128,101,256	–	–	PVT1-374	ENST00000671088.1	5	1191	chr8: 127,854,724–128,101,256	–	–
PVT1-272	ENST00000657844.1	3	609	chr8: 128,045,233–128,101,256	–	–	PVT1-375	ENST00000671092.1	6	1393	chr8: 127,998,358–128,099,834	–	–
PVT1-273	ENST00000657945.1	4	678	chr8: 128,049,408–128,099,677	–	–	PVT1-376	ENST00000671587.1	5	803	chr8: 128,045,317–128,101,256	–	–
PVT1-274	ENST00000658018.1	5	820	chr8: 128,045,317–128,101,250	–	–	**circPVT1**						
PVT1-275	ENST00000658065.1	5	745	chr8: 128,049,409–128,101,256	–	–	–	hsa_circ_0008147	1	11130	chr8:128,009,590–128,020,719	10.1261/rna.035667.112	
PVT1-276	ENST00000658158.1	4	909	chr8: 128,049,152–128,096,763	–	–	circRNA_PVT1	hsa_circ_0009143	2	575	chr8:127,855,155–127,890,998	10.1261/rna.035667.112, 10.1016/j.molcel.2015.03.027, 10.1371/journal.pgen.1003777, 10.1007/s00109-017-1582-9	–
PVT1-277	ENST00000658242.1	2	432	chr8: 127,912,004–127,932,701	–	–	circPVT1	hsa_circ_0001821	1	410	chr8:127,890,589–127,890,998	10.1261/rna.035667.112, 10.1038/nature11928, 10.1016/j.molcel.2015.03.027, 10.1371/journal.pgen.1003777, 10.1007/s00109-017-1582-9	10.18632/aging.103679
PVT1-278	ENST00000658305.1	4	641	chr8: 128,046,599–128,101,256	–	–	–	hsa_circ_0008849	1	855	chr8:128,009,590–128,010,444	10.1261/rna.035667.112	10.3389/fgene.2019.00878, 10.1016/j.canlet.2016.12.006, 10.1093/nar/gkw1201, 10.1186/s13059-017-1368-y
PVT1-279	ENST00000658350.1	6	1402	chr8: 127,997,754–128,101,256	–	–	–	hsa_circ_0135717	2	14014	chr8:127,989,162–128,010,444	10.1016/j.molcel.2015.03.027	–
PVT1-280	ENST00000658429.1	4	760	chr8: 128,039,647–128,101,252	–	–	–	hsa_circ_0085536	3	777	chr8:127,794,533–127,890,998	10.1371/journal.pgen.1003777	–
PVT1-281	ENST00000658556.1	4	1163	chr8: 127,794,550–127,932,708	–	–	–	hsa_circ_0085537	6	1377	chr8:127,794,533–127,989,291	10.1371/journal.pgen.1003777	–
PVT1-282	ENST00000658840.1	2	1738	chr8: 127,997,045–127,999,334	–	–	–	hsa_circ_0085538	7	1490	chr8:127,794,533–128,070,272	10.1371/journal.pgen.1003777	–
PVT1-283	ENST00000659106.1	6	2187	chr8: 127,997,045–128,101,256	–	–	–	hsa_circ_0085539	6	1288	chr8:127,855,155–128,070,272	10.1371/journal.pgen.1003777	–
PVT1-284	ENST00000659326.1	5	1706	chr8: 127,795,196–127,996,681	–	–	–	hsa_circ_0085540	2	579	chr8:127,890,589–127,939,676	10.1371/journal.pgen.1003777	–
PVT1-285	ENST00000659625.1	6	1363	chr8: 127,795,802–128,101,256	–	–	–	hsa_circ_0085541	2	8814	chr8:127,890,589–127,940,456	10.1371/journal.pgen.1003777	–
PVT1-286	ENST00000659666.1	9	1533	chr8: 127,984,004–128,101,252	–	–	–	hsa_circ_0085542	3	880	chr8:127,890,589–127,984,204	10.1371/journal.pgen.1003777	–
PVT1-287	ENST00000659892.1	5	782	chr8: 127,984,171–128,101,256	–	–	–	hsa_circ_0085543	4	1010	chr8:127,890,589–127,989,291	10.1371/journal.pgen.1003777	–
PVT1-288	ENST00000659912.1	9	1744	chr8: 127,795,820–128,101,256	–	–	–	hsa_circ_0085544	5	1123	chr8:127,890,589–128,070,272	10.1371/journal.pgen.1003777	–
PVT1-289	ENST00000660069.1	4	870	chr8: 128048250–128101256	–	–	–	hsa_circ_0085545	1	169	chr8:127,939,508–127,939,676	10.1371/journal.pgen.1003777	–
PVT1-290	ENST00000660122.1	8	1199	chr8: 127,984,004–128,101,256	–	–	–	hsa_circ_0085546	3	600	chr8:127,939,508–127,989,291	10.1371/journal.pgen.1003777	–
PVT1-291	ENST00000660146.1	4	764	chr8: 128,045,285–128,101,256	–	–	–	hsa_circ_0085547	4	713	chr8:127,939,508–128,070,272	10.1371/journal.pgen.1003777	–
PVT1-292	ENST00000660200.1	6	1139	chr8: 127,794,537–127,996,670	–	–	–	hsa_circ_0085548	5	850	chr8:127,939,508–128,096,654	10.1371/journal.pgen.1003777	–
PVT1-293	ENST00000660438.1	13	2450	chr8: 127,795,155–128,017,217	–	–	–	hsa_circ_0085549	6	1124	chr8:127,939,508–128,101,253	10.1371/journal.pgen.1003777	–
PVT1-294	ENST00000660456.1	9	1814	chr8: 127,795,773–128,101,256	–	–	–	hsa_circ_0085550	2	431	chr8:127,983,904–127,989,291	10.1371/journal.pgen.1003777	–
PVT1-295	ENST00000660631.1	12	2161	chr8: 127,794,565–128,101,256	–	–	–	hsa_circ_0085551	3	544	chr8:127,983,904–128,070,272	10.1371/journal.pgen.1003777	–
PVT1-296	ENST00000660659.1	4	673	chr8: 128,045,282–128,101,256	–	–	–	hsa_circ_0085552	4	681	chr8:127,983,904–128,096,654	10.1371/journal.pgen.1003777	–
PVT1-297	ENST00000660781.1	4	854	chr8: 128,045,193–128,101,256	–	–	–	hsa_circ_0085553	2	243	chr8:127,989,162–128,070,272	10.1371/journal.pgen.1003777	–
PVT1-298	ENST00000660896.1	6	1128	chr8: 127,795,928–127,996,670	–	–	–	hsa_circ_0085554	3	380	chr8:127,989,162–128,096,654	10.1371/journal.pgen.1003777	–
PVT1-299	ENST00000660912.1	4	754	chr8: 128,045,204–128,101,256	–	–	–	hsa_circ_0085555	1	113	chr8:128,070,160–128,070,272	10.1371/journal.pgen.1003777	–
PVT1-300	ENST00000661160.1	3	1546	chr8: 127,795,180–127,932,708	–	–	–	hsa_circ_0085556	2	250	chr8:128,070,160–128,096,654	10.1371/journal.pgen.1003777	–
PVT1-301	ENST00000661205.1	7	1449	chr8: 127,795,796–128,101,256	–	–	–	hsa_circ_0085557	3	524	chr8:128,070,160–128,101,253	10.1371/journal.pgen.1003777	–
PVT1-302	ENST00000661391.1	5	924	chr8: 128,045,285–128,101,256	–	–	–	hsa_circ_0085558	2	411	chr8:128,096,518–128,101,253	10.1371/journal.pgen.1003777	–
PVT1-303	ENST00000661625.1	3	1519	chr8: 127,795,346–127,932,701	–	–	–	hsa_circ_0085559	1	274	chr8:128,100,980–128,101,253	10.1371/journal.pgen.1003777	–

Other studies pointed at evaluating the expression of the multiple *PVT1* transcripts by quantifying exons 4A, 4B and 9 in prostate cancer patients [14], even though they did not refer to specific transcript variants. Interestingly, a splicing variant named PVT1b, including exon 1b in place of 1a, was described as having tumour suppressor properties [18, 19] (see the section “lncPVT1 upregulation in human disease and cancer”). According to the Ensembl Genome Browser, multiple transcripts include *PVT1* exon 1b.

Conversely, circular PVT1 variants were detected only by bioinformatics tools [20–23] (Table 1). Functional data are limited to the hsa_circ_0001821 circular RNA (herein referred to as circPVT1), which shares the exon-2 full-length sequence (410 nt) with lncPVT1, and the hsa_circ_0009143 [24]. The latter is overexpressed in cervical cancer and is involved in epithelial–mesenchymal transition (EMT), in which normal polarised epithelial cells transform their phenotype and acquire mesenchymal characteristics and metastasis [24]. circPVT1 derives from a back-splicing event, prompted by a loop structure generated by the presence of Alu repeats flanking exon 2 of *PVT1* [21]. The circular structure makes circPVT1 resistant to exonuclease cleavage, and therefore, highly stable. Indeed, its half-life exceeds 24 h, while lncPVT1 shows a half-life of fewer than 4 h [25].

Although lncPVT1 and circPVT1 are different entities, they are often reported in the literature as involved in the same cellular pathways. This review will introduce their specificities and then focus on their shared pathways, downstream molecular targets and the technical issues encountered to study them as separate entities.

## The *PVT1* locus is frequently amplified and rearranged in human cancer

Multiple *PVT1* genetic variants are described as associated with cancer susceptibility [26–28]. Previous studies mostly documented its involvement in genomic aberrations, e.g., translocations and high copy number amplification, in different malignancies.

Translocations affecting the 8q24 locus are well-documented in multiple myeloma [29], lymphoma [30] and chronic lymphocytic leukaemia [31, 32], and generally result in *MYC* (located 53 Kb upstream of *PVT1* (Fig. 1)) and *PVT1* overexpression; these events are associated with poor prognosis.

Moreover, lncPVT1 has also been reported to be part of fusion transcripts either due to a genomic rearrangement or through trans-splicing events [33, 34]. However, the potential oncogenic roles of these chimeras have not been investigated yet.

8q24 high copy number amplification, in the form of double minute chromosomes or homogeneously staining regions, is described in a series of cancers, from haematological malignancies, such as acute myeloid leukaemia [33, 35] and lymphoma [36], to solid tumours, including gastric cancer (GC) [37], small-cell lung cancer (SCLC) [38], breast cancer [39], medulloblastoma [40], ovarian and endometrial cancers [8, 41, 42], and CRC [43].

The 8q24 genomic amplifications usually cause an increased expression of the embedded oncogenes, particularly *MYC*, even though some exceptions to the amplification-overexpression paradigm are observed [35, 44]. Interestingly, Takahashi et al. demonstrated a stronger correlation between 8q24 copy number gain and *PVT1* expression than the one reported between the genomic amplification and *MYC* [45]. Indeed, a significant amplification of *PVT1* alone was found in some tumour types, suggesting that increased *PVT1* expression may be sufficient to increase MYC levels, which is crucial in tumorigenesis [46, 47]. Increasing literature documented interactions between *MYC* and *PVT1* at both genomic and transcriptional levels, as also discussed in the section “Cell proliferation”. Recent evidence highlighted, in some cancer models (e.g., breast cancer), the role of the *PVT1* promoter in the transcriptional regulation of *MYC*. In detail, Cho et al. identified four *PVT1-*intergenic enhancers increasing *MYC* expression when the *PVT1* promoter is inactive. The latter acts as a DNA boundary element, modulating enhancer–promoter interactions and displaying a tumour-suppressive role [48]. Although the regulatory action of the *PVT1* promoter seems to overcome that of its RNA products, both these elements could contribute to modulating MYC protein levels in a tissue-specific manner. Future studies are needed to clarify the interplay between *PVT1*-mediated transcriptional and post-transcriptional regulation of MYC.

### lncPVT1 upregulation in human disease and cancer

Independently from genomic events, lncPVT1 is upregulated in tumours relative to normal cells in various cancer types, thus representing a good candidate for targeted therapies [49–51].

Interestingly, You et al. reported the hypomethylation of the *PVT1* promoter in several cancer types compared with normal counterparts, suggesting epigenetics as a significant mechanism behind lncPVT1 upregulation [52].

In addition to multiple cellular functions shared between lncPVT1 and circPVT1, which will be discussed later in the review (see the section “Two molecules, same function?”), lncPVT1 is also known to promote angiogenesis, likely by enhancing the expression and secretion of vascular endothelial growth factor (VEGF) [53], and regulating the Wnt/β-catenin axis. lncPVT1 is associated with high cytoplasmic and nuclear β-catenin levels and expression of its CyclinD1 target [54–56]. The upregulation of the Wnt/β-catenin pathway leads to dysregulation of numerous cellular processes, such as cell viability, adhesion, migration, and invasion [57]. Several studies investigated the relationship between *PVT1* and Wnt/β-catenin, all of which focused on the linear isoform [58–60].

Notably, p53 positively regulates the expression level of the PVT1b isoform through its binding to a p53-responsive element, located about 1200 bp downstream the *PVT1* transcriptional start site, between exon 1a and exon 1b, also conserved in mice [18]. The activation of this isoform is stress-dependent, as it is heavily induced after treatment of mouse embryonic fibroblasts and murine lung adenocarcinoma KPR cells with genotoxic or oncogenic stress, respectively [19]. Interestingly, PVT1b activation is accompanied by the specific downregulation of *Myc* transcription, indicating its role as a downstream effector of p53 [19].

This evidence is striking because it underlines the dual behaviour of *PVT1* in cancer, either as an oncogene or as a tumour suppressor gene.

In addition to cancer, the aberrant expression of lncPVT1 has been reported in other pathological conditions. For example, Zhang et al. described lncPVT1 as a therapeutic target for obesity treatment due to its role in preadipocyte differentiation and adipogenesis. Interestingly, they found a significant upregulation of this linear transcript in mature adipocytes compared with preadipocytes, impacting the expression of genes involved in the fatty acid synthesis, transportation and lipogenic transcription [61].

Despite these initial reports, there is still a missing link between the upregulation of lncPVT1 and its causative role in human diseases and cancer development and progression. Moreover, the heterogeneity due to the occurrence of many PVT1 linear isoforms, which hamper specific gene silencing and quantification experiments, represents an issue for investigating the roles of each particular transcript.

### circPVT1 upregulation in cancer and innate immunity

circPVT1 was first described in GC [25], where its expression is upregulated compared with normal gastric tissue. It was subsequently reported as upregulated in several other tumours. Still, its role in carcinogenesis and potential relevance as a diagnostic or prognostic biomarker and as a drug target in cancer remains to be clarified.

Interestingly, circPVT1 expression can be regulated by the interaction between the YAP1 transcriptional cofactor, belonging to the Hippo pathway, and the mutated p53 protein (mut-p53) [62]. YAP1 exerts oncogenic effects by increasing cell proliferation and inhibiting apoptosis. Verduci et al. found a higher expression of circPVT1 in head and neck squamous cell carcinoma patients harbouring *TP53* mutations than in controls [63]. Using siRNA against mut-p53, they observed a downregulation of circPVT1 expression by ~60% 24 h after the transfection. Conversely, no effect on lncPVT1 expression was observed. The authors showed that YAP1 increases circPVT1 expression, acting at both transcriptional (by binding circPVT1 promoter and enhancing its activity) and post-transcriptional (by binding and stabilising circPVT1) levels. This effect is enhanced by mut-p53, which can bind YAP1 and reinforce its interaction with circPVT1. This event, in turn, results in an increased proportion of cells in the cell cycle S and G2 phases and elevated cell proliferation [63].

Finally, many circRNAs, including circPVT1, have been associated with the regulation of innate immunity [64]. Indeed, through the formation of imperfect 16–26-bp RNA duplexes, these highly stable molecules may function as inhibitors of the double-stranded RNA (dsRNA)-activated protein kinase (PKR), which is involved in the innate immune response. Upon viral or bacterial infection, circRNAs are degraded by the endonuclease, RNAse L, resulting in a release and subsequent activation of PKR through autophosphorylation [64]. In addition, individuals affected by systemic lupus erythematosus showed lower levels of many circRNAs in their peripheral blood mononuclear cells, including circPVT1, compared with healthy donors, potentially resulting in an aberrant PKR activation [64].

### Two molecules, same function?

Despite the frequent upregulation of lncPVT1 and circPVT1 in solid tumours and haematological malignancies, their expression levels are poorly correlated [25]. These PVT1 isoforms are transcribed by different promoters [63], therefore, they have to be considered separate transcriptional entities although possibly interconnected.

lncPVT1 is enriched in the nucleus versus the cytosol [19, 65, 66], as observed by subcellular fractionation and subsequent RT-qPCR [19, 65–68], and RNA fluorescence in situ hybridisation [69, 70] in several cancer cell lines. Interestingly, lncPVT1 is described as a chromatin modifier [71]. It has been demonstrated to bind the histone methyltransferase Enhancer of Zeste Homolog-2 (EZH2), a catalytic subunit of polycomb-repressive complex 2 (PRC2), leading to the direct histone methylation of several gene promoters, including the angiopoietin-like 4 (*ANGPTL4*) in cholangiocarcinoma [70] and trophoblast cells [72], the thyroid-stimulating hormone receptor (*TSHR*) in thyroid carcinoma [73], the forkhead box f1 (*FOXF1*) in breast cancer [74], the large tumour suppressor kinase 2 (*LATS2*) in non-small-cell lung cancer (NSCLC) [49], the tumour suppressors p15 and p16 in GC [66], the miR-146a in prostate cancer [75], the miR-200c in melanoma [76], and the miR-200b in cervical cancer [77]. lncPVT1 could also recruit DNMT1 via EZH2 and promote DNA methylation of the miR-18b-5p promoter in gallbladder cancer (GBC) [68]. In liver cancer, instead, lncPVT1 interferes with the recruitment of EZH2 to the *MYC* promoter, thus altering the methylation status and, hence, enhancing its expression [68, 78].

Moreover, lncPVT1 may act as a scaffold for the histone acetyltransferase KAT2A, leading to the final HIF-1α stability increase in nasopharyngeal tumours [71].

Conversely, circPVT1 shows a prevalent cytoplasmic localisation [63, 79–81]. Both lncPVT1 and circPVT1 have been proposed to function as competing endogenous RNAs (ceRNAs) [37, 49, 57, 61]. lncPVT1 functions as a ceRNA by sponging several miRNAs, including miR-186 in GC [82], and miR-186-5p in hepatocellular carcinoma [83]. A similar miRNA-sponging role is described for circPVT1, as for miR-497 in NSCLC [84] and head and neck cancer [63], miR-204-5p in breast cancer [85], miR-125b in NSCLC [79] and GC [25], and miR-145 in CRC [86].

Furthermore, lncPVT1 can directly bind the FOXM1 [87] and MYC [88] proteins to stabilise them post-translationally as well as restrict STAT3 [89] and Lin28 protein degradation by the proteasome machinery [15].

**Fig. 2:**
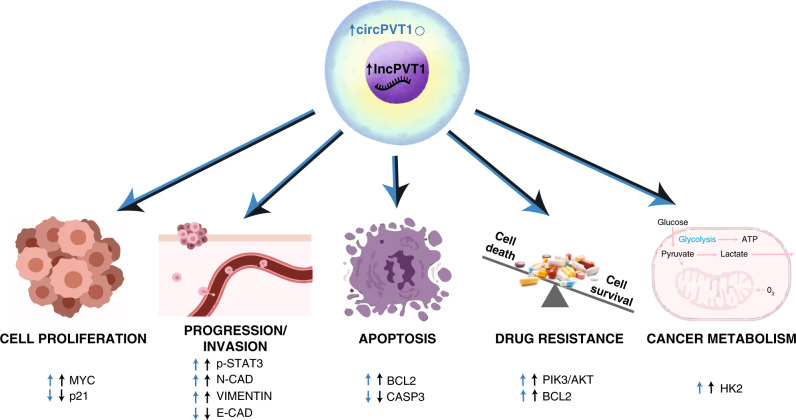
circPVT1 and lncPVT1 shared features and functions in human cancer cells. Pathways regulated by both circPVT1 and lncPVT1 in cancer, as documented in the literature, are shown. Blue and black arrows indicate functions associated with circPVT1 and lncPVT1, respectively. Upward and downward arrows specify the up- and downregulation of the represented molecules, respectively.

circPVT1 and lncPVT1 are largely thought to be involved in the same cellular processes. The main pathways and targets commonly regulated by circPVT1 and lncPVT1 are summarised in the sections “Cell proliferation”, “Oncogenesis and tumour progression”, “Apoptosis”, “Drug resistance”, “Cancer metabolism” and “Clinical impact of lncPVT1 and circPVT1”, and Fig. 2.

However, some of these observations may result from technical issues related to difficulties in discerning the two as separate entities in particular experimental analyses, as discussed in the section “Technical issues for PVT1 quantification and experimental knockdown”.

### Cell proliferation

lncPVT1 and circPVT1 control cell proliferation by regulating target genes such as *MYC* and *CDKN1A* (*cyclin-dependent kinase inhibitor 1A*).

The potential interaction between *MYC* and *PVT1* genes, although widely discussed in the literature, remains controversial. For instance, it is unclear whether these genes may act synergistically, how they are regulated and if *PVT1* linear and/or circular isoforms impact *MYC* transcription and/or translation. In acute lymphoblastic leukaemia (ALL), lncPVT1 increases MYC protein levels with the resulting driver effects on primary tumours [90]. In GC, both circPVT1 and lncPVT1 were described to increase MYC protein levels. circPVT1 facilitates its translation by sequestering let-7b miRNA, whereas, lncPVT1 directly stabilises MYC [25]. lncPVT1, indeed, blocks the phosphorylation of MYC at threonine 58 and prevents its degradation through the ubiquitin–proteasome pathway [47, 88]. Therefore, enhanced lncPVT1 level may increase MYC activity in cancer cells by impairing its turnover. In turn, MYC can act as a *PVT1* transcriptional activator by binding to two E-box elements located at the *PVT1* promoter [9].

Both circPVT1 and lncPVT1 impact the expression of the p21 senescence marker, which is encoded by the *CDKN1A* transcript. By sponging let-7 miRNAs, circPVT1 decreases the level of *CDKN1A* in fibroblast cells [91]. In pancreatic cancer cells, the silencing of lncPVT1 significantly increases the expression level of this tumour suppressor gene, influencing proliferation and migration [92]. Moreover, in NSCLC, lncPVT1 promotes cell proliferation by downregulating p21. This effect was demonstrated by using specific siRNA against lncPVT1 [93]. Similarly, in the Raji Burkitt lymphoma cell line, after lncPVT1 silencing, an increased level of p21 was observed, with a subsequent cell cycle block in G0/G1 phases [94].

### Oncogenesis and tumour progression

In glioblastoma multiforme (GBM), the upregulation of circPVT1 activates, through miR-199a-5p downregulation, the PIK3/AKT pathway, which promotes tumour progression [95]. Interestingly, lncPVT1 in CRC acts as a ceRNA for the tumour suppressor miR-214-3p, leading to increased PIK3/AKT levels, which may cause cancer development [96]. The same effect was observed in human endometrial carcinoma, where lncPVT1 acts through the PVT1/miR-195-5p/FGFR1–FGF2 axis, whose main downstream targets are PIK3/AKT [97].

Moreover, in hepatoblastoma, lncPVT1 overexpression is associated with high levels of p-STAT3, thus promoting proliferation and cancer progression [98]. Accordingly, in oral squamous cell carcinoma (OSCC), circPVT1 sponges miR-125b, which targets the *STAT3* transcript. Therefore, increased circPVT1 levels cause an accumulation of *STAT3*, leading to tumour growth [80].

STAT3 has a well-defined role in cancer development, acting in the VEGFA transcriptional activation, promoting angiogenesis. In GC, a positive feedback loop has been demonstrated between *STAT3* and the lncPVT1 expression: *STAT3* overexpression leads to increased transcription of lncPVT1, which stabilises both STAT3 mRNA and protein in the nucleus. lncPVT1 prevents ubiquitin–proteasomal degradation of phosphorylated STAT3 (p-STAT3), resulting in protein accumulation in the nucleus and activation of the STAT3 signalling pathway [89].

Both lncPVT1 and circPVT1 seem to facilitate cell invasion and metastasis by promoting EMT, losing the adhesion–inhibition capabilities [99]. This phenomenon is mediated by the deregulated expression of key EMT regulators (E-cadherin, N-cadherin and Vimentin), as reported in osteosarcoma, hepatocellular carcinoma, pancreatic cancer, melanoma, oesophageal cancer and cervical cancer [54, 76, 100–103]. Overexpression of lncPVT1 or circPVT1 results in decreased E-cadherin levels (responsible for cell adhesion) and increased expression of N-cadherin and Vimentin (forcing an adhesion-independent phenotype).

### Apoptosis

One of the hallmarks of cancer cells is their capability to escape programmed cell death (apoptosis). Failures in the control of apoptosis may cause tumour initiation, progression and metastasis [104]. Some lncRNAs are negative regulators of apoptosis in tumours [105]. It has been reported that lncPVT1 could inhibit apoptosis in GC tissues through the BCL2 anti-apoptotic factor, having the apoptosis effector CASP3 as a downstream target. When lncPVT1 is upregulated, a simultaneous expression increase of *BCL2* [105].

In osteosarcoma cells, lncPVT1 regulates *BCL2* through miR-195; when lncPVT1 is upregulated, the miR-195 level decreases and *BCL2* transcript increases, resulting in inhibition of apoptosis [106].

circPVT1 also regulates *BCL2* via miRNAs. In NSCLC patients, circPVT1 regulates the miR-497/*BCL2* axis. Indeed, miR-497 shows a binding site at the 3’UTR of the *BCL2* transcript [84].

Finally, in ALL, high levels of circPVT1 sustain BCL2 protein levels, potentially through miR-125 regulation, thus resulting in inhibition of apoptosis. circPVT1 may also force *BCL2* expression to inhibit MYC-mediated apoptosis [90].

### Drug resistance

Both lncPVT1 and circPVT1 were shown to promote drug resistance in several cancer types by affecting, in some cases, the exact molecular targets. For instance, in osteosarcoma, lncPVT1 modulates miR-152 and prevents its binding to the c-mesenchymal–epithelial transition factor (c-MET). This event, in turn, promotes PIK3 activation, inducing drug resistance [107]. The PIK3/AKT pathway is also involved in circPVT1-mediated cisplatin (DDP) resistance in GC. Wang et al. demonstrated that circPVT1 silencing could downregulate the PI3K/AKT signalling through the miR-152-3p/HDGF axis, resulting in decreased DDP resistance and malignancy in GC cells [108].

It has been highlighted that apoptosis and drug resistance are two closely related phenomena in cancer. In GC patients, lncPVT1-mediated upregulation of *BCL2* seems to enhance drug resistance to the 5-fluorouracil (5-FU), leading to a worse prognosis and shorter overall survival (OS) [105]. Involvement in drug response has also been reported in CRC cells, where lncPVT1 upregulation is associated with 5-FU and DDP resistance. This phenomenon is mediated by *BCL2* expression and negative regulation of the apoptotic pathway, influencing BAX and CASP3 pro-apoptotic proteins [109, 110]. In osteosarcoma, circPVT1 upregulation is reported as contributing to doxorubicin (DXR) resistance [111]. Its downregulation in DXR-resistant cell lines resulted in decreased levels of the xenobiotic transmembrane transporters ABCB1 and MRP-1 as well as of BCL2, and increased expression of CASP3 [111].

### Cancer metabolism

Both linear and circular PVT1 can sponge specific miRNAs that modulate *HK2* levels. *HK2* is involved in cellular metabolism, promoting glucose uptake in cancer cells as a carbon source for aerobic glycolysis [112]. High levels of *HK2* are observed in several tumour types and are associated with advanced tumour stage, poor prognosis and metastasis occurrence [113]. A positive correlation between *HK2* and lncPVT1 was detected in osteosarcoma, resulting in a high glucose-uptake rate and subsequent lactate production [114]. *HK2* is a direct target of miR-497, harbouring a candidate-binding site in its 3’UTR. lncPVT1 promotes tumour development by binding miR-497 and blocking its anticancer effects. Moreover, in GBC, lncPVT1 overexpression causes an increase of both *HK2* mRNA and protein by suppressing the miR-143-mediated inhibitory effect [115]. The PVT1/miR-143/*HK2* axis represents the leading target candidate for therapies to regulate cancer metabolism and block tumour progression in GBC.

In OSCC, circPVT1 controls *HK2* levels by sponging miR-106a-5p, contributing to cell growth, metastasis and glycolytic metabolism. Interestingly, the 3’-UTR of *HK2* mRNA displays a binding site for miR-106a-5p that can directly suppress the protein translation. When circPVT1 is upregulated, miR-106a-5p activity is inhibited, leading to increased expression of *HK2*, which promotes cancer development [116].

### Clinical impact of lncPVT1 and circPVT1

The altered expression of lncPVT1 and/or circPVT1 has been associated with tumour progression and poor prognosis in several cancer types (Table 2).

**Table 2:** Clinicopathological significance of lncPVT1 and circPVT1 upregulation in multiple cancer types.

Tumour type	Patient no.	Follow-up (months)	overall survival (OS)	progression-free survival (PFS)/disease-free survival (DFS)	Clinical stage	Lymph node metastasis	Distant metastases	Reference (DOI)
lncPVT1								
Nasopharyngeal cancer	100	125	Poor (*P* < 0.001)	Poor DFS (*P* < 0.010)	na	na	na	10.1038/s41418-019-0381-y
Nasopharyngeal cancer	94	125	Poor (*P* = 0.003)	Poor DFS (*P* = 0.001)	na	na	na	10.1038/s41419-018-0265-y
Nasopharyngeal cancer	20	40	Poor (*P* = 0.040)	Poor DFS (*P* = 0.026)	na	na	na	10.1007/s12253-018-0453-1
Gastric cancer	80	36	Poor (*P* = 0.001)	Poor DFS (*P* = 0.002)	Advanced (*P* = 0.015)	ns	ns	10.1186/s12943-015-0355-8
Gastric cancer	190	85	na	Poor DFS (*P* = 0.002)	ns	ns	Increased (*P* = 0.025)	10.1158/1078-0432.CCR-16-0742
Gastric cancer	111	48	Poor (*P* < 0.001)	Poor DFS (*P* < 0.001)	Advanced (*P* = 0.002)	Increased (*P* = 0.029)	ns	10.4149/314_150825N45
Gastric cancer	200;300*	150;110*	Poor (*P* = 0.008; *P* = 0.042)	na	na	na	na	10.1038/s41388-018-0250-z
Gastric cancer	42	150	Poor (*P* < 0.001)	na	na	na	na	10.1002/jcp.29881
Gastric cancer	17	150	Poor (*P* = 0.032)	na	ns	ns	ns	10.3390/cancers12102995
Gallbladder cancer	55	30	Poor (*P* < 0.001)	na	Advanced (*P* = 0.011)	Increased (*P* = 0.032)	ns	10.1038/s41419-020-03080-x
Gallbladder cancer	66	80	Poor (*P* = 0.002)	na	Advanced (*P* = 0.026)	na	ns	10.1186/s12943-019-0947-9
Non-small-cell lung cancer	nr	120	Poor (*P* = 0.001)	na	na	na	na	10.3892/ol.2019.11237
Non-small-cell lung cancer	105	40	Poor (*P* < 0.001)	Poor PFS (*P* < 0.001)	Advanced (*P* = 0.001)	Increased (*P* = 0.011)	na	10.1158/1535-7163.MCT-15-0707
Non-small-cell lung cancer	108	40	Poor (*P* < 0.001)	Poor PFS (*P* < 0.001)	Advanced (*P* = 0.003)	na	ns	10.1007/s13277-015-4261-x
Non-small-cell lung cancer	31	80	Poor (*P* value nr)	na	Advanced (*P* = 0.017)	Increased (*P* = 0.018)	na	10.1159/000480209
Non-small-cell lung cancer	25	230	Poor (*P* = 0.003)	na	na	na	na	10.2147/OTT.S222898
Non-small-cell lung cancer	82	60	Poor (*P* < 0.050)	na	na	Increased (*P* = 0.001)	na	Yang et al. [117] (PMC4230094)
Small-cell lung cancer	120	60	Poor (*P* = 0.024)	na	Advanced (*P* < 0.001)	Increased (*P* < 0.001)	Increased (*P* < 0.001)	Huang et al. [118] (PMC5126345)
Epithelial ovarian cancer	231	90	Poor (*P* = 0.020)	Poor PFS (*P* = 0.002)	Advanced (*P* < 0.001)	ns	na	10.20892/j.issn.2095-3941.2017.0174
Epithelial ovarian cancer	73;129*	200	Poor (*P* = 0.0012; *P* < 0.001)	Poor PFS (*P* < 0.001; *P* < 0.001)	na	na	na	10.1158/1078-0432.CCR-16-1402
Ovarian cancer	40	60	Poor (*P* value nr)	na	na	na	na	10.1016/j.biopha.2018.06.112
Colorectal cancer	112	60	Poor (*P* = 0.019)	na	Advanced (*P* = 0.001)	Increased (*P* = 0.015)	Increased (*P* = 0.007)	Ping et al. [109] (PMC5801353)
Colorectal cancer	210	72	Poor (*P* < 0.001)	Poor DFS (*P* < 0.001)	Advanced (*P* < 0.001)	Increased (*P* < 0.001)	na	10.1177/1724600818777242
Colorectal cancer	62	60	Poor (*P* = 0.040)	na	Advanced (*P* < 0.001)	Increased (*P* = 0.005)	Increased (*P* = 0.002)	10.2147/CMAR.S260537
Colorectal cancer	239;75*	60	Poor (*P* = 0.007; *P* = 0.039)	na	na	na	na	10.1186/s12943-020-01277-4
Colorectal cancer	164	180	Poor (*P* = 0.0101)	na	Advanced (*P* = 0.002)	Increased (*P* = 0.0079)	ns	10.1038/bjc.2013.698
Osteosarcoma	26	60	Poor (*P* < 0.050)	na	na	na	na	10.18632/oncotarget.13012
Osteosarcoma	46	72	Poor (*P* < 0.050)	na	Advanced (*P* < 0.001)	na	na	10.1016/j.bbrc.2017.06.024
Diffuse large B‐cell lymphoma	286	84	Poor (*P* < 0.001)	Poor PFS (*P* < 0.001)	na	na	na	10.1002/cac2.12073
Multiple myeloma	128	42	Poor (*P* = 0.012)	Poor PFS (*P* = 0.002)	Advanced (*P* = 0.012)	na	na	10.1177/1533033820935496
Diffuse glioma	98	170	Poor (*P* < 0.0001)	na	Advanced (*P* < 0.001)	na	na	10.18632/oncotarget.20226
Uveal melanoma	80	80	Poor (*P* = 0.009)	na	ns	na	na	10.1371/journal.pone.0189675
Renal cell carcinoma	528	120	Poor (*P* = 0.001)	Poor DFS (*P* = 0.001)	Advanced (*P* < 0.050)	na	Increased (*P* = 0.017)	10.18632/oncotarget.19743
Oesophageal squamous cell carcinoma	52	100	Poor (*P* < 0.001)	Poor DFS (*P* = 0.011)	Advanced (*P* = 0.001)	na	na	10.18632/oncotarget.15878
Oesophageal squamous cell carcinoma	156	120	Poor (*P* = 0.004)	na	Advanced (*P* = 0.043)	na	na	10.1186/s12943-019-1064-5
Oesophageal carcinoma	50	40	Poor (*P* < 0.050)	Poor DFS (*P* < 0.050)	na	na	na	10.1002/1878-0261.12555
Cervical cancer	127	nr	Poor (*P* = 0.030)	na	na	na	na	10.1371/journal.pone.0156274
Cervical cancer	90	60	Poor (*P* = 0.015)	na	Advanced (*P* < 0.01)	na	na	10.1111/apm.12555
Pancreatic cancer	30	100	Poor (*P* = 0.008)	na	ns	Increased (*P* = 0.004)	na	10.7150/jca.37959
Breast cancer	209	300	Poor (*P* < 0.050)	na	na	na	ns	10.1038/s41388-018-0310-4
Breast cancer	110	60	Poor (*P* = 0.003)	na	Advanced (*P* = 0.002)	Increased (*P* = 0.023)	Increased (*P* = 0.023)	10.1016/j.bbrc.2017.09.005
Hepatocellular cancer	214	124	ns	Poor DFS (*P* = 0.021)	Advanced (*P* < 0.050)	na	na	10.3892/ol.2014.2730
Hepatocellular cancer	89	50	Poor (*P* = 0.0104)	Poor DFS (*P* = 0.004)	Advanced (*P* = 0.007)	na	na	10.1002/hep.27239
circPVT1								
Gastric cancer	187	85	Good (*P* < 0.001)	Good DFS (*P* = 0.002)	ns	ns	ns	10.1016/j.canlet.2016.12.006
Head and neck squamous cell carcinoma	106;263*	72;210*	Poor (*P* = 0.050)	na	na	na	na	10.1186/s13059-017-1368-y
Osteosarcoma	80	60	Poor (*P* = 0.002)	na	Advanced (*P* = 0.044)	na	Increased (*P* = 0.038)	10.7150/ijbs.24360
Osteosarcoma	48	60	Poor (*P* = 0.005)	na	Advanced (*P* = 0.008)	na	Increased (*P* = 0.009)	10.1111/jcmm.15215
Osteosarcoma	36	50	Poor (*P* = 0.028)	na	na	na	na	10.1111/cas.14787
Non-small-cell lung cancer	90	60	Poor (*P* < 0.050)	na	Advanced (*P* = 0.007)	ns	na	10.1016/j.biopha.2018.12.007
Non-small-cell lung cancer	96	100	Poor (*P* = 0.020)	na	Advanced (*P* = 0.003)	ns	na	10.1177/0300891620941940
Non-small-cell lung cancer	8	60	Poor (*P* = 0.002)	na	Advanced (*P* < 0.001)	Increased (*P* = 0.001)	na	10.1186/s13046-021-01976-w
Non-small-cell lung cancer	104	60	Poor (*P* = 0.011)	na	Advanced (*P* = 0.027)	na	na	10.1016/j.biopha.2020.109828
Colorectal cancer	64	60	Poor (*P* < 0.001)	na	Advanced (*P* = 0.002)	na	na	10.1016/j.bbrc.2019.03.121
Hepatocellular carcinoma	70	60	Poor (*P* = 0.024)	na	Advanced (*P* = 0.029)	Increased (*P* = 0.004)	na	10.1242/bio.043687
Ovarian cancer	nr	200	na	Poor DFS (*P* = 0.005)	na	na	na	10.7150/jca.52234
Breast cancer	99	60	Poor (*P* = 0.022)	na	Advanced (*P* = 0.012)	ns	na	10.2147/OTT.S180850
Medullary thyroid cancer	28	48	Poor (*P* < 0.050)	na	na	na	na	10.1186/s13046-021-01964-0

*nr* not reported, *na* not analyzed, *ns* not significant.

*Two patient cohorts investigated.

In particular, elevated expression levels of lncPVT1 predict poor prognosis and worse clinicopathological characteristics in both solid and haematological malignancies, resulting in a decrease of OS, progression-free survival (PFS) and/or disease-free survival (DFS). Indeed, as reported in Table 2, the lncPVT1 upregulation in tumour tissues is associated with an advanced clinical stage and the presence of lymph node and distant metastases. Similar results were obtained when analysing the clinical impact of circPVT1 overexpression in different solid tumours: it predicted a poor OS and was related with an advanced clinical stage and, when analysed, with the occurrence of lymph node and distant metastases (Table 2). The only exception is GC. In this malignancy, high circPVT1 expression was associated with a good prognosis, likely due to its positive correlation with the tumour suppressor miR-125, which blocks the cell cycle at the G0/G1 phase, seeming to promote apoptosis, and inhibits tumour growth and invasion [25].

In summary, both lncPVT1 and circPVT1 might serve as effective prognostic biomarkers for multiple tumour entities.

### Technical issues for PVT1 quantification and experimental knockdown

As lncPVT1 and circPVT1 share the same genomic sequence corresponding to lncPVT1 exon 2, technical approaches capable of discriminating between them are required to understand their individual biological roles.

In this context, we analysed the sequences of primers and siRNAs used to quantify and silence either lncPVT1 or circPVT1 across the literature.

**Fig. 3:**
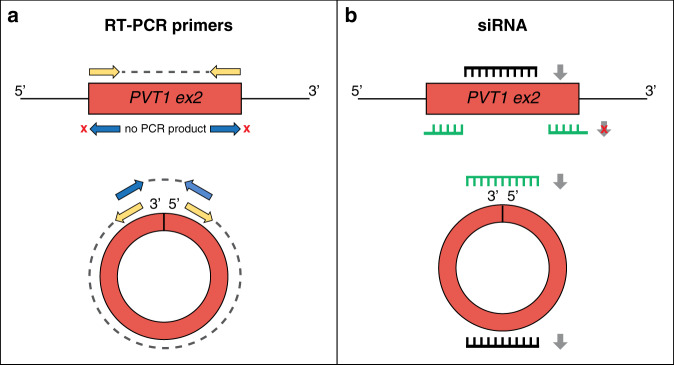
Technical challenges in PVT1 transcript quantification and silencing assays. **a** Convergent primers (yellow arrows) designed on lncPVT1 exon 2 give rise to PCR products from both linear and circular isoforms (grey dashed line), whereas divergent primers (blue arrows) amplify a specific PCR product from circPVT1. **b** A siRNA designed on lncPVT1 exon 2 (black line) silences both lncPVT1 and circPVT1. The specific silencing of circPVT1 can be achieved using a siRNA targeting its back-splicing junction (green line). Grey arrows = transcript silencing.

Primers for RT-qPCR assays are often designed within exon 2 of *PVT1* with a convergent orientation. If not preceded by RNase R digestion of the linear transcripts, this approach introduces a bias in quantification due to the primer pair annealing to both the circular and linear isoforms, as shown in Fig. 3a. Conversely, divergent primers on exon 2 allow the selective amplification of circPVT1, not requiring preventive digestion of the linear isoform and avoiding issues due to a partial efficiency of this step (Fig. 3a).

Similarly, in knockdown experiments, siRNAs specifically designed on *PVT1* exon 2 will not allow a specific inhibition of one of the two isoforms, introducing a bias in evaluating the results, as shown in Fig. 3b. Thus, in a significant fraction of the published papers, it is not clear if the knockdown-related effects are attributable to lncPVT1 or circPVT1 or the result of both being simultaneously silenced. This problem can be overcome by placing the siRNA on the circPVT1 back-splicing junction (Fig. 3b) and a linear splicing junction for lncPVT1.

These technical issues question many published studies claiming specific functions for one of the two isoforms. In particular, the results suggest that both isoforms are involved in the same cellular processes. Thus, more studies are needed to clarify whether the observed effects result from a synergistic action of the two *PVT1* isoforms or from technical artefacts.

## Conclusions

The *PVT1* gene has been widely investigated for its roles in cancer. However, the discovery of multiple linear and circular isoforms disclosed its multifaceted activity, with several aspects still to be clarified.

lncPVT1 and circPVT1 have to be considered two distinct entities, possibly sharing certain biological functions and having separate roles in cancer.

The molecular mechanisms behind their involvement in cancer initiation and progression have started to be disentangled. Of note, both transcripts might serve as prognostic biomarkers, and their possible connection with *MYC* highlights their possible role as targets of future therapies. More work is needed to clarify their potential interactions and roles as distinct transcript entities in cancer, mainly due to technical issues on the distinction between linear and circular isoforms in many published studies.

## Supplementary information

AJ Checklist

## Data Availability

Not applicable.
